# The Role of Apoptosis and Oxidative Stress in a Cell Spheroid Model of Calcific Aortic Valve Disease

**DOI:** 10.3390/cells13010045

**Published:** 2023-12-25

**Authors:** Colin W. Coutts, Ashley M. Baldwin, Mahvash Jebeli, Grace E. Jolin, Rozanne W. Mungai, Kristen L. Billiar

**Affiliations:** Biomedical Engineering Department, Worcester Polytechnic Institute, Worcester, MA 01609, USAgejolin@wpi.edu (G.E.J.);

**Keywords:** valvular interstitial cell, calcific aortic heart valve disease, apoptosis, oxidative stress, antioxidant

## Abstract

Calcific aortic valve disease (CAVD) is the most common heart valve disease among aging populations. There are two reported pathways of CAVD: osteogenic and dystrophic, the latter being more prevalent. Current two-dimensional (2D) in vitro CAVD models have shed light on the disease but lack three-dimensional (3D) cell–ECM interactions, and current 3D models require osteogenic media to induce calcification. The goal of this work is to develop a 3D dystrophic calcification model. We hypothesize that, as with 2D cell-based CAVD models, programmed cell death (apoptosis) is integral to calcification. We model the cell aggregation observed in CAVD by creating porcine valvular interstitial cell spheroids in agarose microwells. Upon culture in complete growth media (DMEM with serum), calcium nodules form in the spheroids within a few days. Inhibiting apoptosis with Z-VAD significantly reduced calcification, indicating that the calcification observed in this model is dystrophic rather than osteogenic. To determine the relative roles of oxidative stress and extracellular matrix (ECM) production in the induction of apoptosis and subsequent calcification, the media was supplemented with antioxidants with differing effects on ECM formation (ascorbic acid (AA), Trolox, or Methionine). All three antioxidants significantly reduced calcification as measured by Von Kossa staining, with the percentages of calcification per area of AA, Trolox, Methionine, and the non-antioxidant-treated control on day 7 equaling 0.17%, 2.5%, 6.0%, and 7.7%, respectively. As ZVAD and AA almost entirely inhibit calcification, apoptosis does not appear to be caused by a lack of diffusion of oxygen and metabolites within the small spheroids. Further, the observation that AA treatment reduces calcification significantly more than the other antioxidants indicates that the ECM stimulatory effect of AA plays a role inhibiting apoptosis and calcification in the spheroids. We conclude that, in this 3D in vitro model, both oxidative stress and ECM production play crucial roles in dystrophic calcification and may be viable therapeutic targets for preventing CAVD.

## 1. Introduction

Calcific aortic valve disease (CAVD) affects approximately 2–4% of people over the age of 65 and is characterized by the thickening of fibrotic leaflets as well as the formation of calcific nodules [1]. While there are several types of treatments, the current standard of care for CAVD is to replace the valve through surgery. This treatment, however, has short-term risks such as the dangers of surgery, and long-term risks such as morbidity and increased complications attributed to anticoagulation as well as the need for reoperation because of the limited lifespan of prosthetic valves [2]. Transcatheter aortic valve replacement (TAVR) is another option currently offered for patients with excess operative risk, and it is becoming a more popular option due to the procedure being significantly less invasive compared with surgical aortic valve replacement; however, TAVR suffers from complications as well [3]. The overall problem remains, as there are no medical therapies for CAVD as alternatives to surgery [2]. Originally, CAVD was presumed to be a degenerative disease that affects older populations since their valves have been opening and closing every second of their lives [1]. This process, however, is now known to be a more active cell-driven process, which provides hope in finding a way of inhibiting calcification by modifying cellular processes [1].

There are two types of cell-driven processes that lead to the calcification of the valve. The first is osteogenic calcification, which occurs when the valvular interstitial cells (VICs) adopt an osteoblast-like phenotype, which will produce an osteoid matrix [4]. While this is the better-understood cause of calcification, it is only responsible for 13% of all cases; the other process is dystrophic calcification, which is responsible for 83% of all cases. [4]. Dystrophic calcification involves VIC activation in a myofibroblast-like phenotype and subsequent calcification through an apoptosis-dependent mechanism [4]. Yip et al. found, specifically, that apoptosis led to calcification on stiffer substrates, and that it can be attributable to a local contraction of the cell layer resulting from increased cytoskeletal tension, which is then released on aggregation [5]. This was tested through constrained collagen gels seeded with VICs where they observed a significant increase in the number of apoptotic cells [5]. Dystrophic calcification of VICs has also been shown to be dependent upon the extracellular matrix (ECM) composition of the substrate on which they are cultured [6].

While the mechanism for VICs undergoing apoptosis is not currently known, it has been shown by multiple research groups that apoptosis plays a role in calcification in 2D in vitro experimental systems [7]. One example comes from Walker et al., who seeded VICs at high densities into culture dishes and then supplemented them with TGF-β1 [8]. VICs exhibited markers for the myofibroblast phenotype and formed cell aggregates. Apoptosis was then observed within the middle of the aggregates, which began calcifying. In an in vitro model developed by Fisher et al., aggregates were formed rapidly with a combination of mechanical stretch and TGF-β1, apoptosis occurred in the center, and subsequently calcific nodules formed [9]. Through using Z-VAD, a pan-caspase inhibitor that prevents apoptosis though irreversibly binding to the catalytic site of caspase proteases, the group observed a significant reduction in the number of calcific nodules, further indicating the importance of apoptosis to the calcification process [9]. This method, however, had limitations associated with the consistency and reproducibility of the 2D spheroids. Using micro-contact printing, our group was able to consistently reproduce cell aggregation with myofibroblastic markers, apoptosis, and calcium accumulation [10]. The method was found to be highly reproducible, with 70% of spheroids staining positive for calcium (via Alizarin Red S) after one week in culture. Mineralized calcium-positive nanoparticles were found within the VIC aggregates along with positive staining for the apoptotic pathway marker caspase [10]. This study demonstrated that TGF-beta treatment is not necessary for the calcification of the aggregates and likely plays more of a role in the aggregation process. Yet all of the aforementioned studies have the limitation that they studied the calcification of 2D aggregates which lack the 3D cell–cell and cell–matrix interactions found in vivo.

Several groups have developed 3D models to study CAVD in vitro. These models typically culture isolated (non-aggregated) VICs within hydrogels made from polymers such as photo-crosslinked hyaluronic acid (HA) and/or gelatin, and they utilize osteogenic media to initiate calcification. Butcher and colleagues cultured human aortic VICs within 3D HA hydrogels with adjustable stiffness and found that constructs with an initially valve leaflet layer-like stiffness significantly promote the myofibroblastic phenotype [11]. Similarly, Anseth and colleagues encapsulated porcine aortic VICs within MMP-degradable PEG-based hydrogels and demonstrated that the gel modulus has an influence on VIC morphology and calcification, whereas stiffer gels lead to higher levels of calcium deposition [12]. Aikawa and colleagues developed a 3D system using a hydrogel made from photo-crosslinked HA and gelatin and found that VICs encapsulated into the 3D hydrogels only activate into myofibroblasts when exposed to exogenous environmental cues, and they did not observe apoptotic-driven calcification [13]. Grande-Allen and colleagues used a layered filter paper-based cell culture system to determine the relative amount of fibrillar collagen within a 3D construct that regulates the osteogenic capacity of VICs [14]. As the osteogenic medium is utilized in these 3D models, they provide little insight into the dystrophic pathway.

As 2D models are unable to mimic the complex valve environment, and 3D models employ non-aggregated cells and osteogenic media, there is a need for a 3D model in which dystrophic calcification can be studied. Interestingly, Roosens et al., in their attempt to engineer healthy valve tissues by generating valvular interstitial cell spheroids, found that the 3D spheroids calcified when grown in standard media. They were able to overcome this “limitation” by supplementing the media with ascorbic acid, which they postulated acts as an antioxidant, protecting the cells against oxidative stress-induced cell damage [15]. Antioxidants have been shown to have protective effects against apoptosis, which, as previously stated, has been shown to be integral to calcification in 2D models [16]. This VIC spheroid model is a potentially powerful system in which to study the mechanisms of dystrophic calcification in 3D culture.

The goal of this work is to develop a 3D dystrophic calcification model based on cell microspheres. We hypothesize that, as with 2D cell-based CAVD models, apoptosis is integral to the calcification process. We model the cell aggregation observed in CAVD by creating porcine valvular interstitial cell spheroids in non-adherent microwells and treating the spheroids with multiple different antioxidants with known protective effects against oxidative stress. Spheroids were also stained to visualize calcific nodules, live and dead cells, and apoptosis.

## 2. Materials and Methods

### 2.1. Cell Culture

Porcine aortic valvular interstitial cells (PAVICs) were extracted from porcine hearts obtained from a local abattoir (Blood Farm, Groton, MA, USA) within three hours of tissue harvest as per published protocols and used in experiments from passages 4–9 [17]. Cell cultures were maintained in a complete cell medium containing DMEM (Gibco, Waltham, MA, USA), 5% fetal bovine serum (Gibco), and 1% antibiotic-antimycotic (Gibco). All cell cultures were maintained at 37 °C and 10% CO_2_.

### 2.2. Spheroid Formation and Media Supplementation

Spheroids were formed using Microtissues^®^ negative molds with 300 μm diameter x 800 μm deep wells based on Roosens et al. [15]. Negative molds were filled with 3% *w*/*v* agarose solution made with SeaKem^®^ LE Agarose in DPBS (Corning, New, York, NY, USA); once they were solidified, one million PAVICs were evenly distributed in 200 μL to fill the 256 wells per mold (Supplementary Figure S1). Each mold was cultured in a separate dish or well and complete media or complete media supplemented with antioxidants was replenished every 2–3 days.

To determine the effects of antioxidants on calcification, three different antioxidants were supplemented into the culture media. Ascorbic acid as well as two antioxidants with similar effects were chosen: Trolox and Methionine. These antioxidants were specifically chosen for their known protection against reactive oxygen species (ROS), which play important roles in cell death, differentiation, and signaling while having differential effects on inducing ECM compared to AA. Initial concentrations used for testing were chosen based on literature showing the effectiveness of the antioxidants at 250 μm for AA (L-Ascorbic acid 2-phosphate sesquimagnesium salt hydrate, Millipore Sigma A8960, Burlington, MA, USA), 100–200 μm for Methionine (L-Methionine, Millipore Sigma M9625), and 1 mM for Trolox ((±)-6-Hydroxy-2,5,7,8-tetramethylchromane-2-carboxylic acid, Sigma Aldrich 238813, Burlington, MA, USA) [18,19]. Trolox was not soluble in DMEM and was, therefore, dissolved in 70% ethanol and added to DMEM with the ethanol being no more than 0.5% of the final volume.

### 2.3. Calcium Staining

Von Kossa staining was performed to visualize calcium buildup per Deegan et al. [20]. Spheroids were fixed in 10% Formalin (Sigma Aldrich) and then put under UV light (365 nm, 115 V, 0.68 Amps) for 20 min in 1% aqueous silver nitrate (MP Biomedicals). Excess silver nitrate was removed using 5% sodium thiosulfate (Thermo Fisher Scientific, Waltham, MA, USA) for 5 min. Spheroids were counterstained using 0.1% nuclear fast red (Polysciences Inc., Warrington, PA, USA) for 3 min. Spheroids were then washed and imaged in 70% ethanol. Calcium buildup was quantified by optimizing the threshold of each spheroid individually (ImageJ, Java 8, open source https://imagej.net/software/imagej/, (accessed on 20 December 2023)), leaving the calcific nodules as black areas and the surroundings as white (Supplementary Figure S2). These calcific nodule areas were measured to determine the overall area of the calcified regions using a particle analyzer. This area was then divided by the total area of the spheroid, measured by tracing the edges of the spheroid, to calculate the percentage of calcification area per area of the spheroid. Transmitted light images were taken using 10× and 20× magnification, as specified in the image captions, using a Zeiss microscope (Jena, Germany).

### 2.4. Cell Viability

Cell viability was measured using AlamarBlue^TM^ (Thermo Fisher Scientific). Cells were washed with PBS 1x (Corning), and the media was replenished with 10% AlamarBlue^TM^ and allowed to culture for 8 h. The measurement of fluorescence signals was conducted at an excitation wavelength of 530–560 nm and an emission wavelength of 590 nm [21]. Cell viability was also imaged with Live/Dead staining with calcein/propidium iodide.

### 2.5. Measuring and Blocking Apoptosis

Apoptosis in spheroids was blocked by supplementing DMEM with 20 uM Z-VAD for 5 days before seeding in agarose molds per Cirka et al. [10]. Z-VAD-FMK, also known as carbobenzoxy-valyl-alanyl-aspartyl-[O-methyl]-fluoromethylketone, was added at the same concentration to replenish the media for the duration of the experiment. To ensure Z-VAD was properly blocking apoptosis, the cells were stained for cleaved caspase (CellEvent Caspase 3/7—Invitrogen, Waltham, MA, USA) and then imaged using a fluorescent microscope (Zeiss). Staining for caspase 3 and caspase 7 covers the major executioner caspases [22]. Using ImageJ, the spheroids were outlined, and the integrated density of the gray value was measured; then a small circle next to the spheroid was created to measure the background grey value (Supplementary Figure S3). The corrected total cell fluorescence per area was then calculated with the following equation: (integrated density) − (area of spheroid) × (mean background)/area.

### 2.6. Inducing Oxidative Stress

To induce oxidative stress on the cells, culture media was supplemented with hydrogen peroxide. The concentrations of hydrogen peroxide tested on the cells were based on the literature on inducing stress on fibroblasts; these studies indicate that a range between 10 μm and 1 mM is effective [23]. Concentrations in which cells were seen to have reduced proliferation as well as visible dead cells (through Live/Dead staining) without killing the majority of the population were considered effective [23]. To determine ideal concentrations for our cells, VIC monolayers were incubated with concentrations of H_2_O_2_ in DMEM from 1 μm to 750 μm once they reached roughly 80% confluency. The cells were incubated for 24 h followed by a Live/Dead (Invitrogen) stain to determine the overall viability of the cells.

### 2.7. Statistical Analysis

Descriptive statistics are reported as the mean and standard deviation. A one-way ANOVA on ranks was performed due to the non-normality of the data sets and post hoc pairwise comparisons were completed using Dunn’s test, and *p* < 0.05 was considered statistically significant. All statistics were performed on replicates of at least 10 spheroids unless otherwise specified.

## 3. Results

**Figure 1:**
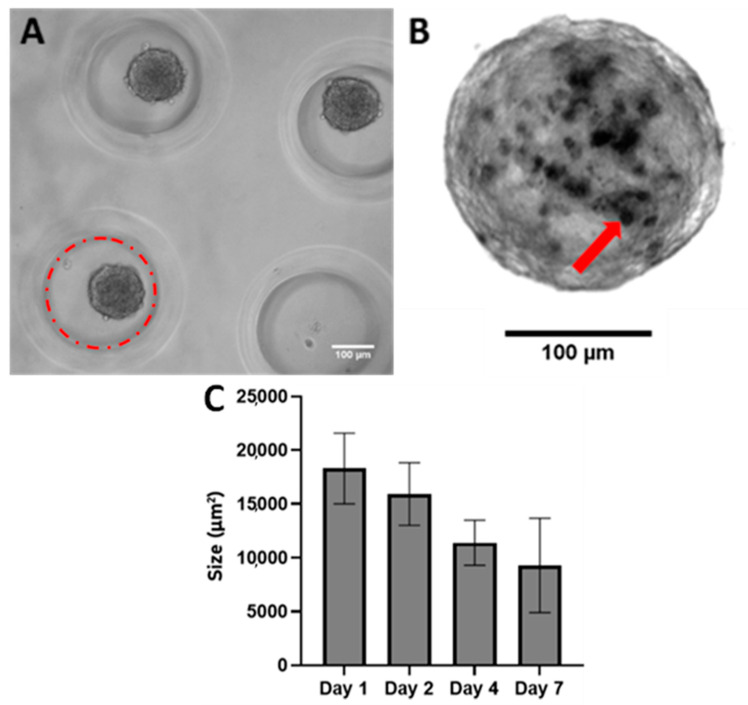
(**A**) Phase (10×) image of control day 2 porcine aortic VIC spheroids formed in non-adherent agarose mold. Each mold is seeded with one million VICs. The red circle indicates the outline of a single well in the mold, with the darker circles being formed spheroids. Scale bar = 100 μm. (**B**) Calcified Spheroid: Phase (20×) image of Von Kossa stain showing calcification on a day 4 porcine aortic VIC spheroid. The red arrow points to a calcific nodule, which became black once stained. Scale bar = 100 μm. (**C**) Change in spheroid size over the course of seven days in culture.

Spheroids were formed in non-adherent wells after as little as two days (Figure 1A). These spheroids gradually decreased in size as the culture period continued, as was found in Roosens et al. (2017), and were presumably due to the compaction of the cells producing their own ECM (Figure 1B). A Live/Dead stain was performed on the wells after seeding to ensure that the vast majority of cells put into the model were alive. On day four after seeding into the wells, spheroids had an average diameter of 160 μm. Calcium could be visualized within the spheroids as early as day four and gradually increased over time. Based on these findings, imaging of the spheroids was performed on day four after deposits could be visualized and then on day seven to visualize the progression after one full week, which was the typical length of a 2D calcification experiment in the literature [10].

At times, spheroids merge together to form double and triple spheroids. Within these larger spheroids, multiple cores of calcification and dead cells can be seen. The dual core is clearly observed through both Von Kossa stains (Supplementary Figure S4) and Live/Dead stains (Supplementary Figure S5). This observation points to the importance of the microenvironment within the spheroids as well as ECM composition in determining the health of the cells.

### 3.1. Effect of Ascorbic Acid Supplementation on Spheroids

**Figure 2:**
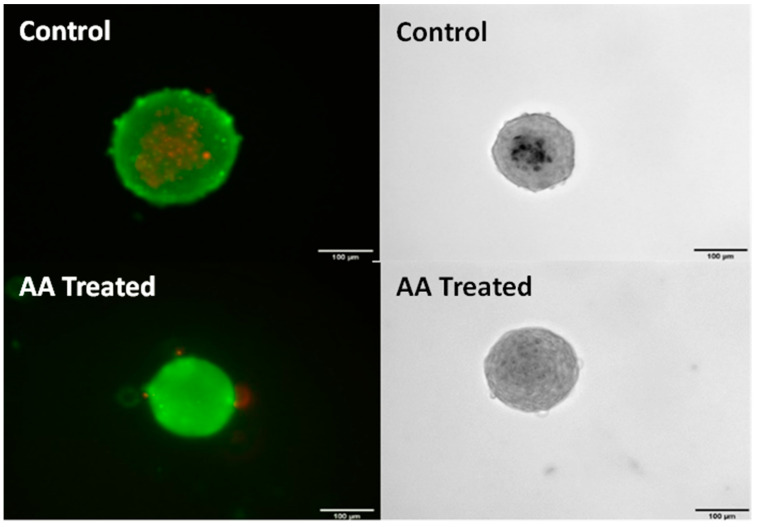
Control spheroids (**top**) and ascorbic acid-supplemented spheroids (**bottom**) after four days in culture. Fluorescent (**right**) with live (green)/Dead (red) stain and 10× phase (**left**). Passage 7 porcine aortic VICs were used. Supplementation with 250 μm ascorbic acid almost completely inhibited cell death and prevented calcification. Scale bar = 100 μm.

To determine if ascorbic acid has the same anti-calcification effects on our cell line as previously observed, spheroids were supplemented with 250 μm from day zero. Von Kossa stains revealed that supplementation of AA was in fact able to prevent calcification as seen in Figure 2. Cells of the same passage were also stained with Live/Dead, showing that AA also prevented cell death, which was observed in the center on non-treated spheroids.

### 3.2. Effect of Blocking Apoptosis with Z-VAD

**Figure 3:**
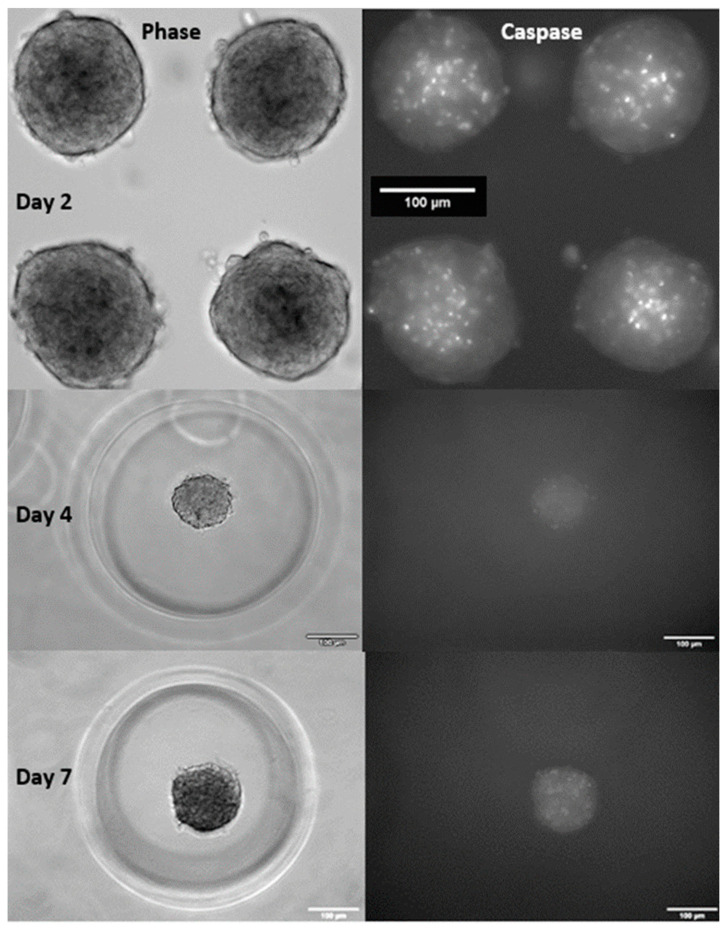
Z-VAD supplementation reduces apoptosis: Day 2 Control (**top**), Day 4 (**middle**), and Day 7 (**bottom**) spheroids 10× phase (**left**) and with caspase stain (**right**). Passage 9, day 4 porcine aortic VIC spheroids treated with 20µM caspase inhibitor Z-VAD for five days prior to creating the spheroids and for the duration of the experiment. Scale bar = 100 μm.

Previous experiments have shown dense regions of apoptosis centered in the core of control spheroids (Figure 3). The mean corrected total cell fluorescence per area for these spheroids was 937 ± 74 mean fluorescence intensity (MFI). To determine if apoptosis is integral to calcification in 3D spheroids as it has been demonstrated in 2D aggregates, apoptosis was blocked using Z-VAD followed by Von Kossa staining. The implementation of Z-VAD for 5 days culture in 2D before seeding spheroids in micro-molds resulted in a mean corrected total cell fluorescence per area of 106 ± 46 MFI (Figure 3). So, while Z-VAD significantly reduces the apoptosis in spheroids, it is important to note that there are still spots of apoptosis that occur, resulting in potential nucleation sites for calcium nodule formation.

By day seven, the apoptosis significantly increased in spheroids treated with Z-VAD with a mean corrected total cell fluorescence (CTCF) per spheroid of 286 ± 62. This would result in increasing nucleation sites for calcium deposits (Figure 3). Overall, this shows that Z-VAD is effective as a caspase inhibitor in the cell spheroids, minimizing but not fully eliminating programmed cell death.

**Figure 4:**
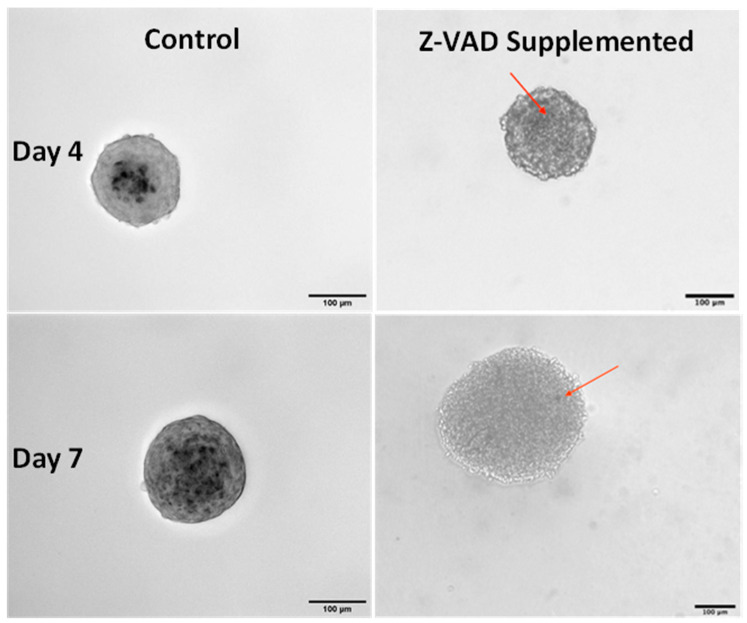
Z-VAD supplementation inhibits calcification. Images (20×, transmitted light) of passage 9, day 4 (**top**), and day 7 (**bottom**) of porcine aortic VIC spheroids stained with Von Kossa. Control spheroids (**left**) show pronounced calcification as indicated by dark nodules. In contrast, spheroids treated with Z-VAD show only slight calcification (indicated with a red arrow). Scale bars = 100 μm.

The relative lack of Von Kossa staining on day-four spheroids with Z-VAD indicates minimal calcification with a few calcium nodules present (Figure 4 (left)). This extent of calcification is noticeably less than calcium formation on non-treated spheroids (Figure 4 (right)). Due to Z-VAD not entirely blocking apoptosis, nucleation sites still form. Over the course of seven days, this gradual slow apoptosis builds up, resulting in the calcific spots shown in Figure 4.

### 3.3. Determination of Antioxidant Concentrations in 2D Culture

In the 2D monolayer tests, we determined that the ideal concentrations of H_2_O_2_ in DMEM for the purpose of this study were 250 μm and 500 μm, as these levels provided clear effects of the induced oxidative stress without killing the majority of the cells as determined by Live/Dead staining (Supplementary Figure S6). These concentrations of H_2_O_2_ were then used on cells in monolayers that were pretreated with ascorbic acid, Trolox, or methionine for 24 h. H_2_O_2_ was added for 24 h, and AlamarBlue^TM^ was added for the final 8 h. This resulted in an AlamarBlue^TM^ signal, as a value that showed the ascorbic acid and Trolox fully protecting against the induced oxidative stress at 250 μm and 500 μm, respectively (Supplementary Figure S7). Methionine was able to offer some protection from the induced stress at both tested concentrations but was most protective at 200 μm. This provided enough evidence that these antioxidants provide significant protection against oxidative stress to be used on the 3D spheroid model.

### 3.4. Antioxidant Effects in 3D Spheroids

**Figure 5:**
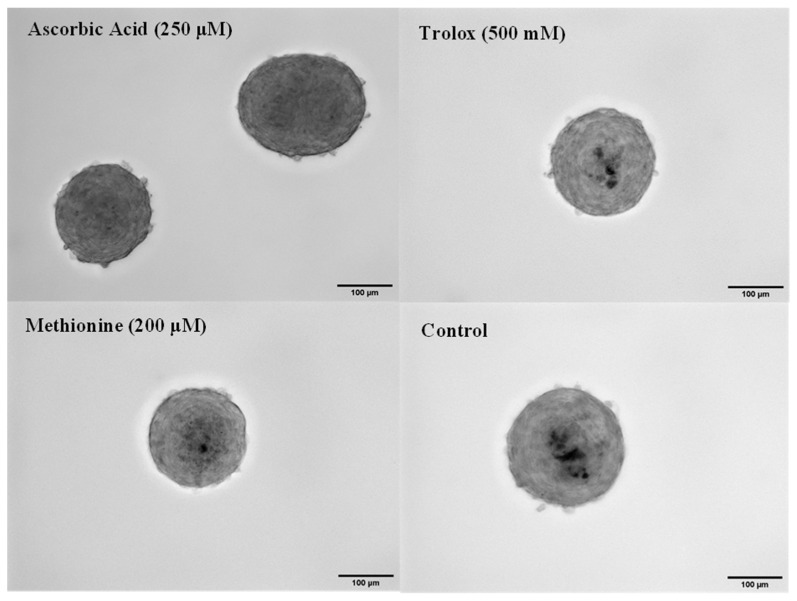
Calcium stain on antioxidant-supplemented spheroids on day 4: Images (20×) of passage 4 porcine aortic VIC spheroids on day 4 with Von Kossa staining for various antioxidant treatments. Scale bar = 100 μm.

After determining the effective concentrations of the antioxidants to protect against H_2_O_2_-induced oxidative stress, they were supplemented in the media fed to spheroids from day one of the culture. On day four, Von Kossa staining shows that calcification is significantly reduced in spheroids treated with the AA and Trolox compared to the control (Figure 5). Image analysis reveals that AA and Trolox had 0.07% ± 0.05% and 0.7% ± 0.5% areas calcified, respectively. Compared to the control, which had 3.01% ± 1.5% calcification, this was a significant decrease. Methionine also had a slight reduction in calcification buildup compared to the control at 1.6% ± 0.8% area calcified. These changes have been attributed to the ability of the antioxidants to protect against oxidative stress and, therefore, reduce apoptosis.

**Figure 6:**
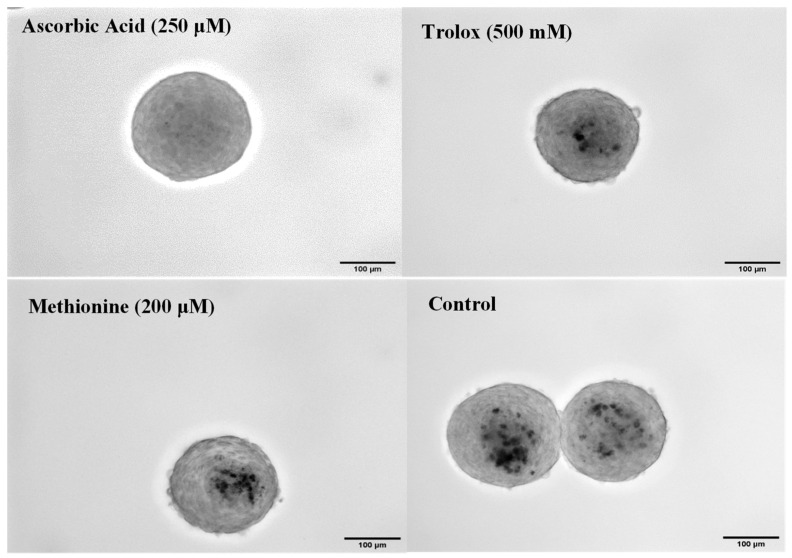
Calcium stain on antioxidant-supplemented spheroids on day 7: Phase image (20×) of passage 4 porcine aortic VIC spheroids on day 7 with Von Kossa staining for various antioxidant treatments. Scale bar = 100 μm.

**Figure 7:**
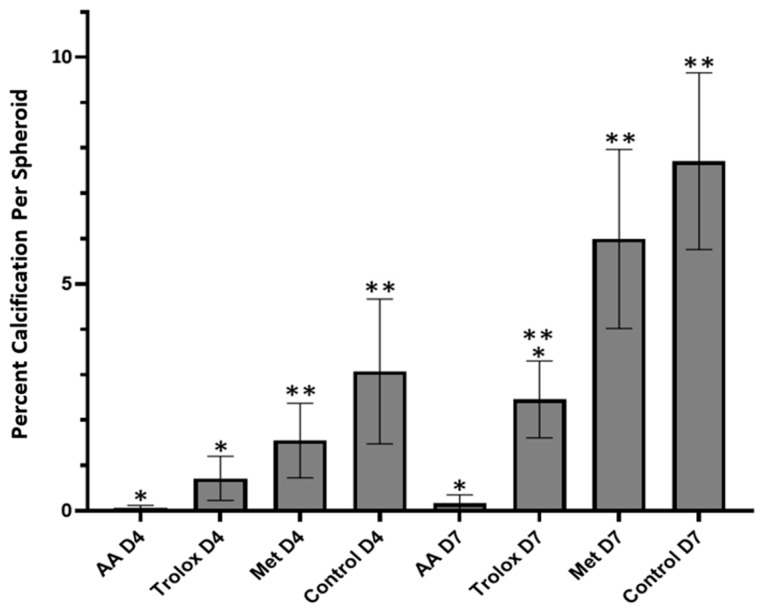
Quantification of results from antioxidant treatment: Percent of calcification per total area of spheroids treated with different antioxidants (AA 250 μm, Trolox 500 μm, Methionine 200 μm) on days 4 and 7. Asterixs (*) represent a significant difference from the same-day control group (no antioxidants), and double asterixs (**) represent a significant difference from the same-day AA-treated group (*p* < 0.05; one-way ANOVA on ranks with Dunn’s test post hoc pairwise comparisons).

By day seven, Von Kossa staining revealed that the calcification progressed similarly to day four (Figure 6 and Figure 7). At this time point, however, the calcification buildup in Trolox had become much more prevalent at 2.5% ± 0.8%, while AA was still able to prevent calcium buildup nearly completely at 0.2% ± 0.2%. Both were significantly less than the control, which had 7.7% ± 1.9% of the total area as calcium positive. Methionine had a large calcium buildup in the core of the spheroid, resulting in 6.0% ± 1.9%, which was slightly less than the control. Spheroids on days four and seven were also measured for their total areas to see if there were any significant differences in size depending on which antioxidant was supplemented. No significant differences in the area of the spheroids on the same day were found; however, there was a small reduction in the area between days 4 and 7 (*p* > 0.05; Supplementary Figure S8).

## 4. Discussion

In this work, we developed a 3D valvular interstitial cell microsphere model of CAVD. This model was based on the work of Roosens et al., who reported undesirable calcification of VIC spheroids for tissue engineering applications that could be abrogated by ascorbic acid treatment [15]. Using this model, we demonstrate that apoptosis is integral to the calcification process, which is in agreement with 2D cell aggregate models of dystrophic calcification. Our data indicate that oxidative stress plays a role in the calcification process and that other effects of ascorbic acid, likely related to ECM production, are also critical in limiting dystrophic calcification in this model. In comparison to the progression of calcification in vivo, this model as well as 2D models in the literature calcify significantly faster. Our results are consistent with the rate of calcification observed by Roosen et al., who visualize calcium deposits after only three days [15]. This enhanced rate may be due to the forced aggregation of a large mass of cells, which interact to activate the dystrophic pathway. One advantage of this accelerated process is that it allows for the rapid assessment of the effect of potential therapeutic compounds on calcification.

One of the well-studied parts of the dystrophic pathway in 2D vascular calcification models is programmed cell death. Proudfoot et al. revealed that apoptosis occurs prior to the onset of calcification, and apoptotic bodies can produce calcium in a crystallized form [24]. The same group found that the inhibition of apoptosis with the caspase inhibitor Z-VAD is capable of reducing calcification nodules by roughly 40% [25]. This was recapitulated in 2D CAVD models by subsequent groups, who found that by using Z-VAD to block apoptosis in VIC monolayers, there was a significant reduction in the number of calcific nodules [7,9]. Inhibiting apoptosis by an alternative agent, M50054, also significantly decreases calcium nodule formation in porcine aortic VICs [26]. To see if increasing programmed cell death would produce the opposite result, Proudfoot et al. stimulated apoptosis in nodular cultures using anti-Fas IgM, and found that there was a 10-fold increase in calcification [24]. Fujita et al. took a different approach, where they added dead cells to the culture and found that necrotic areas became surrounded by calcium deposits [27]. Mathieu et al. found that apoptotic bodies derived from plasma membrane are rich in ectonucleotidases and promote the nucleation and formation of spheroid mineralized microparticles, which are the basic unit of the mineralized material formed in CAVD [28]. Further, Galeone et al. demonstrated that the pro-apoptotic cytokine TRAIL is found to be expressed in human calcified aortic valves but not in normal healthy ones [29]. With numerous studies showing the importance of programmed cell death in the formation of calcium nodules, we decided to incorporate similar studies to see if this finding would be recapitulated in 3D. By blocking apoptosis through a caspase inhibitor Z-VAD, we found a great reduction in calcium formation. While Z-VAD was effective at significantly reducing the amount of apoptosis occurring in the spheroids, it was not able to completely prevent apoptosis and, thus, did not completely block calcification. The lack of efficacy in the 3D spheroids is believed to be due to the lack of penetration of the Z-VAD into the center of the spheroids; however, the diffusion of metabolites does not appear to be limited in our spheroids as cell death was not observed in spheroids treated with ascorbic acid (Figure 2) or in deeper areas of aggregated spheroids (Supplementary Figure S4).

Another important focus of research on CAVD has been on the role of the ECM proteins in valvular calcification. Hutson et al. found that CAVD is associated with layer-specific alterations in collagen architecture by comparing diseased and healthy human aortic valves; they specifically point to the disorganization of the valve’s ECM as a hallmark of CAVD [30]. Fondard et al. also found that alterations of the ECM, with a focus on the disorganization of collagen bundles and the fragmentation of elastic fibers, are significantly higher in aortic stenosis and aortic regurgitation when compared with controls [31]. Further, Eriksen et al. performed an analysis of explanted calcified valves and found that the total collagen content in valves with CAVD was significantly lower than that found in normal valves [32]. Together, these findings indicate that collagen is disrupted and/or diminished in CAVD. To test if a decrease in collagen content could cause CAVD (rather than occur due to calcification), Rodriguez et al. partially removed collagen from porcine leaflets and observed an increase in VIC proliferation and apoptosis relative to non-collagen-depleted leaflets, which remained relatively quiescent [33].

As ascorbic acid induces collagen biosynthesis [34], it is possible that AA prevents calcification in our 3D spheroid model by inducing collagen production in addition to its antioxidant activity. In 2D studies, VICs cultured on collagen and fibronectin have been shown to be resistant to calcification relative to cells cultured on tissue culture plastic; this resistance to mineralization holds up even upon treatment with mineralization-inducing growth factors such as BMPs and TGF-β1 [35]. To determine if collagen has a direct effect on calcification in our 3D model, we supplemented the cell suspension in a subgroup of samples with 1µL of 0.05 mg/mL collagen before spheroid formation. Von Kossa staining patterns on repeat trials of this experiment were highly variable and yielded no statistically significant differences after four or seven days when compared to non-supplemented control spheroids (Supplementary Figure S9). It is unclear if the addition of collagen produces variable results or if the incorporation of collagen was itself variable. Similarly, in an attempt to create calcium-rich engineered tissues, Leach and colleagues incorporated cell-secreted ECM into mesenchymal stromal cell spheroids and found an increase in calcium accumulation, whereas incorporating exogenous collagen led to a slight decrease; however, neither difference was significant relative to untreated controls even after 21 days of culture in osteogenic media [36]. Taken together, these studies show an important correlation between ECM components on calcification, yet the mechanisms are still yet to be elucidated.

The final focus of this study revolved around the role of oxidative stress and antioxidants in the dystrophic pathway. Many different cardiovascular disorders and diseases have been associated with a state of oxidative stress where the expression levels of reactive oxygen species-producing enzymes are up-regulated [37]. In these diseases, which include endothelial dysfunction, hypertension, and cardiac remodeling, the activity and expression of antioxidant mechanisms are down-regulated [37]. Elevated oxidative stress has also been observed to be correlated with the severity of aortic stenosis (AS) [38]. Recent studies support the concept that reactive oxygen species (ROS), which can be produced by inflammation, play an important role in the development of the early cellular and extracellular changes associated with aortic valve stenosis [39]. Miller et al. found that superoxide and H_2_O_2_ levels were significantly increased near calcified regions in explanted valves from patients with established aortic stenosis [40]. This is likely one of the reasons for AA’s anti-calcification effects, since it acts as an electron donor to reduce the production of ROS and, therefore, helps prevent DNA damage, oxidation of amino acid residues, as well as lipid peroxidation [41]. These findings support our selection of antioxidants to test, as they were shown to have protective effects against oxidative stress-induced through H_2_O_2_.

Based on these findings, we aimed to determine if there is a correlation between antioxidant protection against ROS and calcification in our 3D model. Trolox and Methionine were chosen for their known protection against ROS and also their differential effects on inducing ECM production as AA. Trolox and AA are in the same family as they are vitamin E and vitamin C, respectively. Trolox, unlike AA, does not increase the formation of collagen in fibrous tissue, and it has been shown to be more potent in promoting regenerative capacity in comparison to AA [16]. Both are capable of significantly reducing intracellular oxidative stress. Methionine, on the other hand, is an essential amino acid in humans and is largely involved in the production of cysteine, which is used to build proteins in the body [42]. This reagent has been shown to have strong effects on improving cellular oxidative balance as well as mediating oxidative stress where it targets ROS directly by being oxidized to methionine sulfoxide [42]. We found that supplementation in culture media from day zero of spheroid formation had a direct impact on the amount of calcium buildup. While AA was effective at completely preventing calcification, Trolox and methionine had a less pronounced effect but were both able to significantly reduce the percentage of calcium per area compared to the control. These data indicate that antioxidant activity alone is not enough to prevent calcium accumulation, but they reveal a promising avenue for therapeutic research. To that point, despite this strong evidence that ROS are involved in CAVD, oral antioxidant treatments in atherosclerosis have been largely unsuccessful [43].

Future studies are needed to examine the role of the ECM within spheroids and how it affects calcium nodule formation. One possible explanation of our data could be that oxidative stress leads to remodeling of aortic valves, causing changes in the ECM that lead to apoptosis, and thus results in calcium nucleation sites, which build up over time. Since AA and Trolox maintained cell viability despite induced oxidative stress, but only AA was able to fully prevent any calcium buildup, it would be beneficial to determine the ECM composition of those spheroids. Comparing this with the ECM of controls as well as Methionine, which had some prevention of calcification, could provide insight as to what specific components have calcium-preventative capabilities. Focusing on specific ECM components could also be beneficial, with a specific focus recommended for collagen, which is a major component of heart valve ECM. Supplementation of spheroids with collagen could be completed to see the impact on calcium buildup. Another possible study would be to reintroduce the ECM of spheroids supplemented with AA. Doing so could be useful for determining if the main preventative qualities of AA in calcium buildup come from its effect on the ECM or rather its antioxidant properties. It could also be beneficial to co-stain spheroids and image them using a confocal microscope to characterize the co-localization of the regions of calcification, apoptosis, and collagen. To gain further insight into merged spheroids, we would recommend performing an experiment to determine when and under what specific conditions spheroids will merge and then imaging them at different time points after merging. Lastly, to better mimic an in vivo environment, spheroids should be seeded into a stretchable model to incorporate mechanical stresses.

## 5. Conclusions

Through these experiments, we were able to show that the spheroid model produces dystrophic calcification and is, therefore, a promising 3D model for studying CAVD. Overall, this study was able to recapitulate findings from previous 2D studies, revealing that apoptosis is also integral to dystrophic calcification in 3D. We were also able to show a correlation between calcium formation and oxidative stress through the use of antioxidant supplementation in a spheroid culture medium. By imaging merged spheroids, we were also able to show a direct correlation between the microenvironment of the spheroids, as the calcium deposits are only present in the center of a formed spheroid. These findings lead us to believe that calcification is the result of multiple factors, including oxidative stress as well as cell interactions with extracellular matrix components (or lack thereof).

## Data Availability

All images and data referenced in this work can be accessed via the Harvard Dataverse at: https://doi.org/10.7910/DVN/ZORZER (accessed on 20 December 2023).

## Supplementary Materials

The following supporting information can be downloaded at: https://www.mdpi.com/article/10.3390/cells13010045/s1, Figure S1: Aggregate Formation: Method of forming aggregates through filling a negative mold with non-adherent agarose, then filling the mold with one million cells evenly suspended in DMEM. Cells will then settle into the bottom of the 256 wells and form aggregates in roughly 70% of the wells; Figure S2: Calcium Quantification: Measurement of calcium nodules using ImageJ with phase image (left) and thresholded and binarized image (right). Scale bar = 100 μm; Figure S3: Caspase Quantification: To measure corrected total cell fluorescence aggregates were outlined, measured, and then also measured outside of the aggregate to determine background using ImageJ. Scale bar = 100 μm; Figure S4: Calcium Stain of Merged Aggregates: Phase (10×) image of Von-Kossa stain on two (left) and three (right) merged day 4 porcine aortic VIC aggregates. Aggregate formation can still be seen in merged aggregates with the circular cell orientation around calcific nodules in both images. Red circles show the approximate locations where the individual aggregates’ microenvironments remain within the merged aggregates. Scale bar = 100 μm; Figure S5: Live/Dead Stained Merged Aggregate: Live (green)/Dead (red) stain of a singular (top) and two merged day 2 porcine aortic VIC aggregates (bottom). The image reveals a gap in dead cells between the merged aggregates (represented by the red dotted line) where as there is a single core of cell death in the sole aggregate. Scale bar = 200 μm; Figure S6: Oxidative stressing of VICs in 2D with Hydrogen Peroxide: Determining Hydrogen Peroxide concentration to induce stress to PAVICs using Live(green)/Dead(red) stain. Each row represents four replicates at each concentration (n = 4). A pronounced increase in dead cells was observed between 250 μM and 500 μM without killing a majority of the cells. Scale bar = 100 μm; Figure S7: AlamarBlueTM corrected total cell fluorescence (CTCF) quantification of cell viability after hydrogen peroxide-induced oxidative stress on two 2D cell culture replicates. Results show that a Trolox concentration of 500 μM and ascorbic acid concentration of 250 μM protected the cells against the induced stress. Methionine provided less protection against induced stress with the most protection at a concentration of 200 μM. Negative control (red dashed line) is H_2_O_2_ without any antioxidant and positive control (blue dotted line) control without H_2_O_2_; Figure S8: Aggregate projected area as a function of day and antioxidant treatment. No significant differences in area of spheroids of the same day were found; however, there was a small reduction in area between day 4 and 7 (*p* > 0.05); Figure S9: Collagen Supplementation Has Variable Effects. Cell spheroids were supplemented with 1 μL of 0.05 mg/mL collagen into the cell suspension before spheroid formation then stained with Von Kossa for calcium. Repeat trials of this experiment revealed variable incorporation of collagen and variable sized aggregates. There was no statistically significant difference in cell calcification after 4 or 7 days when compared to non-supplemented control spheroids; Figure S10: Confocal Images taken of PAVIC 3D spheroid. Spheroids were stained with Propidium Iodide (Top) and Hoechst (Bottom) to better understand the location of the cells as well as where apoptosis is occurring. From the Propidium Iodide staining we were able to confirm that cellular death is occurring in the center of the aggregates with little to no cell death along the outer edges. The Hoechst staining revealed that the cells were rather evenly spread around the spheroid with less live cells in the center of the spheroid.
