# Supplementary material for: The Role of Apoptosis and Oxidative Stress in a Cell Spheroid Model of Calcific Aortic Valve Disease
Source: Cells. 2023 Dec 25;13(1):45. doi: 10.3390/cells13010045 (PMC10778193; doi:10.3390/cells13010045)
Supplement: Supplementary file 1 [file cells-13-00045-s001.zip › cells-2749071-supplementary.pdf]

Supplementary Information for

The Role of Apoptosis and Oxidative Stress in Cell Spheroid Calcification

Colin Coutts, Ashley Baldwin, Mahvash Jebeli, Grace Jolin, Rozanne Mungai, Kristen Billiar

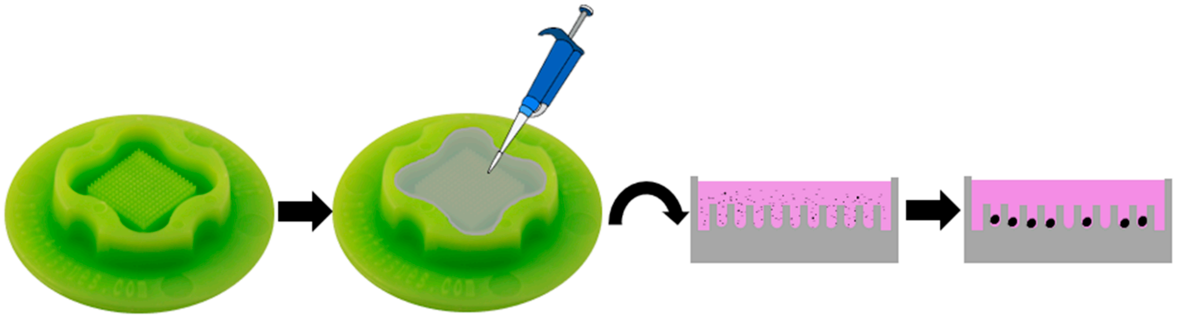

Figure S1. Aggregate Formation: Method of forming aggregates through filling a negative mold with non-adherent agarose, then filling the mold with one million cells evenly suspended in DMEM. Cells will then settle into the bottom of the 256 wells and form aggregates in roughly 70% of the wells.

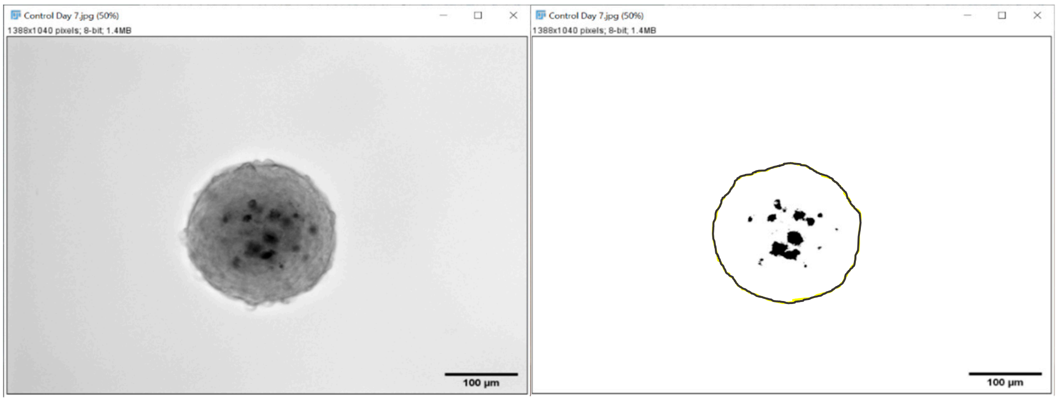

Figure S2. Calcium Quantification: Measurement of calcium nodules using ImageJ with phase image (left) and thresholded and binarized image (right). Scale bar = 100μm.

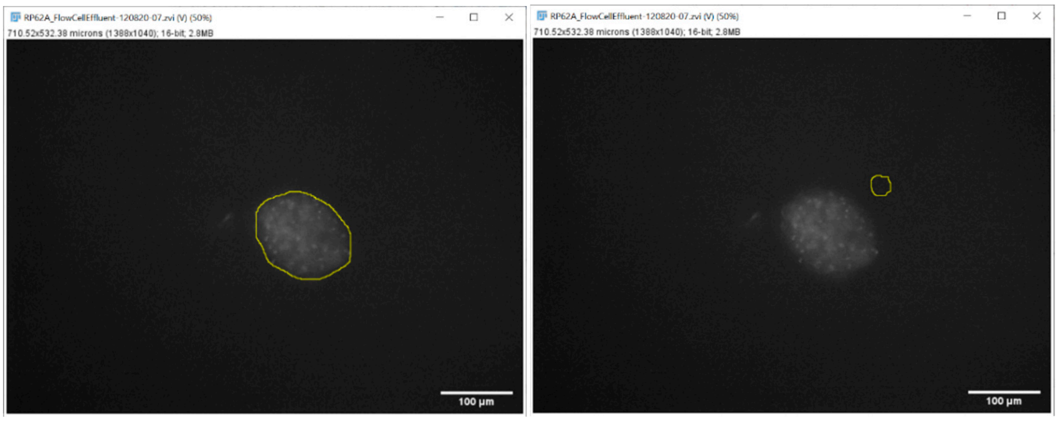

Figure S3. Caspase Quantification: To measure corrected total cell fluorescence aggregates were outlined, measured, and then also measured outside of the aggregate to determine background using ImageJ. Scale bar = 100µm.

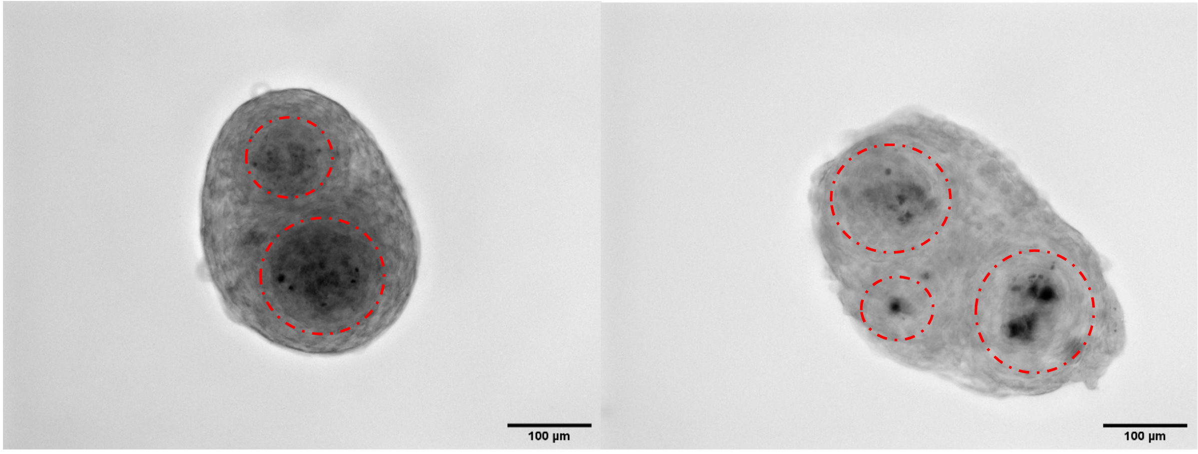

Figure S4. Calcium Stain of Merged Aggregates: Phase (10x) image of Von-Kossa stain on two (left) and three (right) merged day 4 porcine aortic VIC aggregates. Aggregate formation can still be seen in merged aggregates with the circular cell orientation around calcific nodules in both images. Red circles show the approximate locations where the individual aggregates' microenvironments remain within the merged aggregates. Scale bar = 100µm.

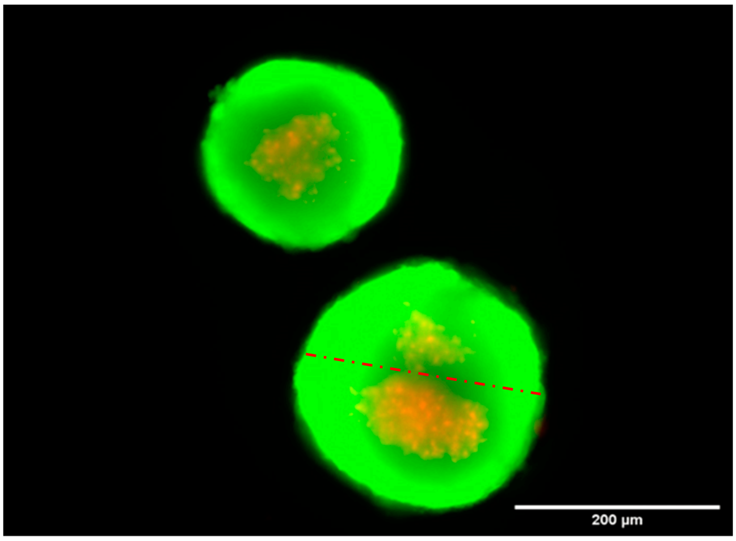

Figure S5 Live/Dead Stained Merged Aggregate: Live (green)/Dead (red) stain of a singular (top) and two merged day 2 porcine aortic VIC aggregates (bottom). The image reveals a gap in dead cells between the merged aggregates (represented by the red dotted line) where as there is a single core of cell death in the sole aggregate. Scale bar = 200µm

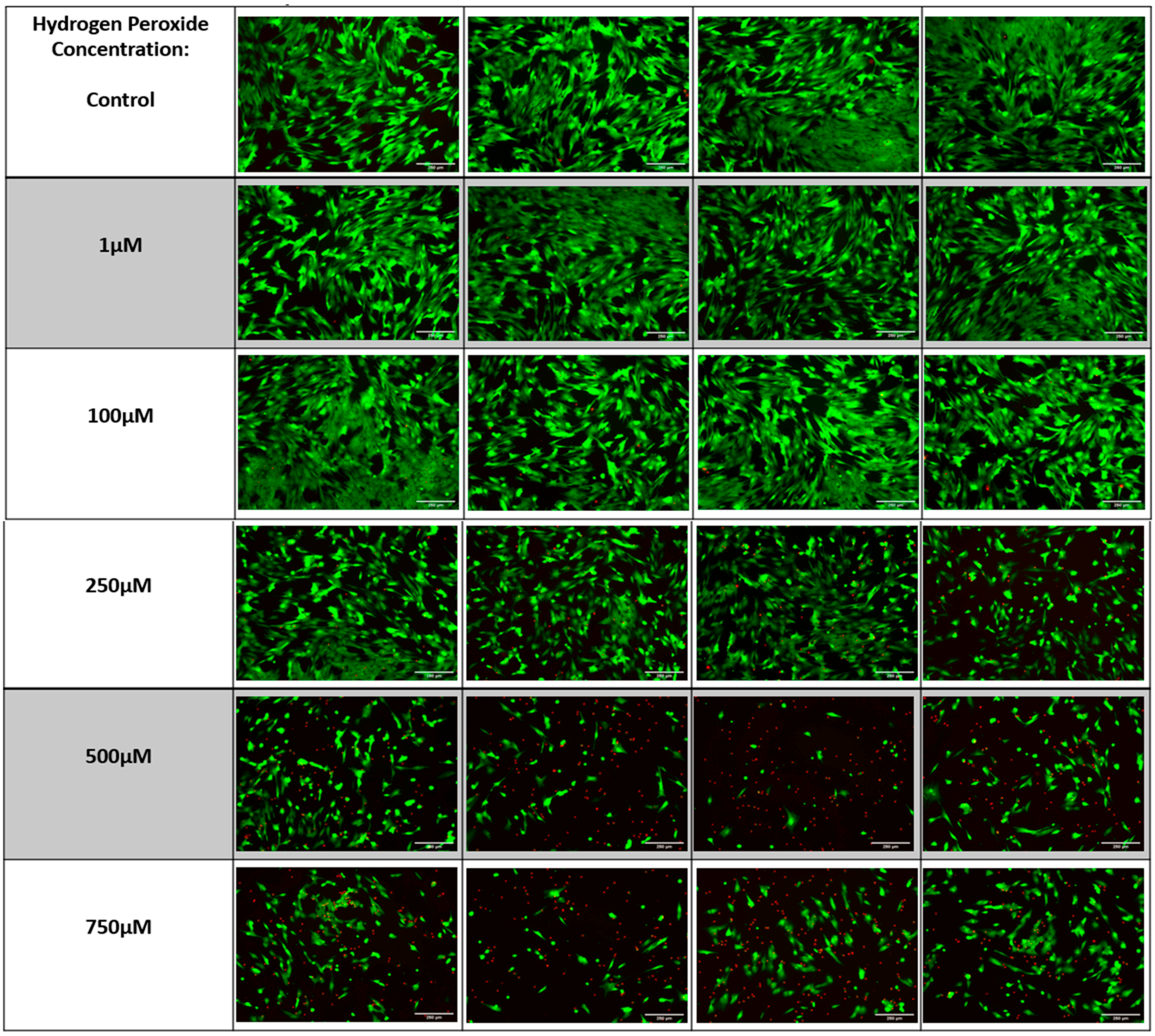

Figure S6. Oxidative stressing of VICs in 2D with Hydrogen Peroxide: Determining Hydrogen Peroxide concentration to induce stress to PAVICs using Live(green)/Dead(red) stain. Each row represents four replicates at each concentration (n=4). A pronounced increase in dead cells was observed between 250µM and 500µM without killing a majority of the cells. Scale bar = 100µm

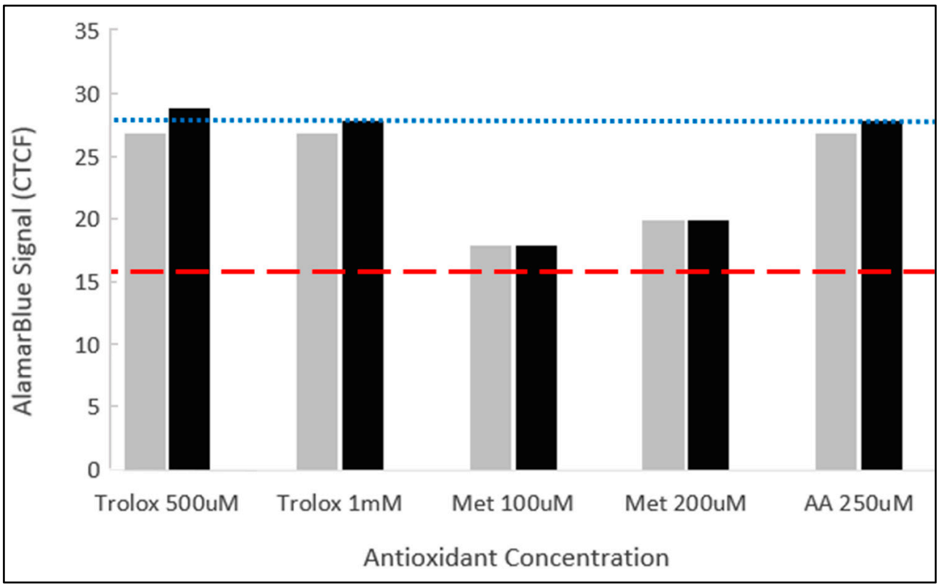

Figure S7. AlamarBlue™ corrected total cell fluorescence (CTCF) quantification of cell viability after hydrogen peroxide-induced oxidative stress on two 2D cell culture replicates. Results show that a Trolox concentration of 500µM and ascorbic acid concentration of 250µM protected the cells against the induced stress. Methionine provided less protection against induced stress with the most protection at a concentration of 200µM. Negative control (red dashed line) is H_2O_2 without any antioxidant and positive control (blue dotted line) control without H_2O_2.

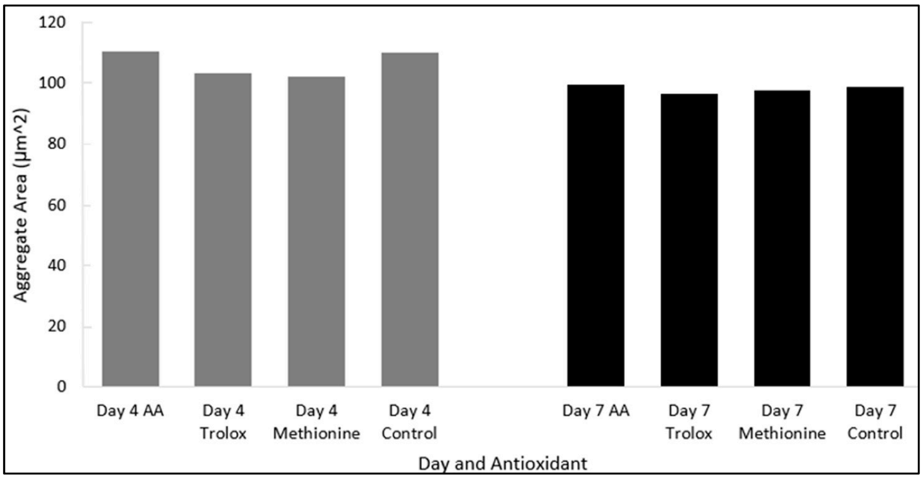

Figure S8. Aggregate projected area as a function of day and antioxidant treatment. No significant differences in area of spheroids of the same day were found; however, there was a small reduction in area between day 4 and 7 ($p>0.05$).

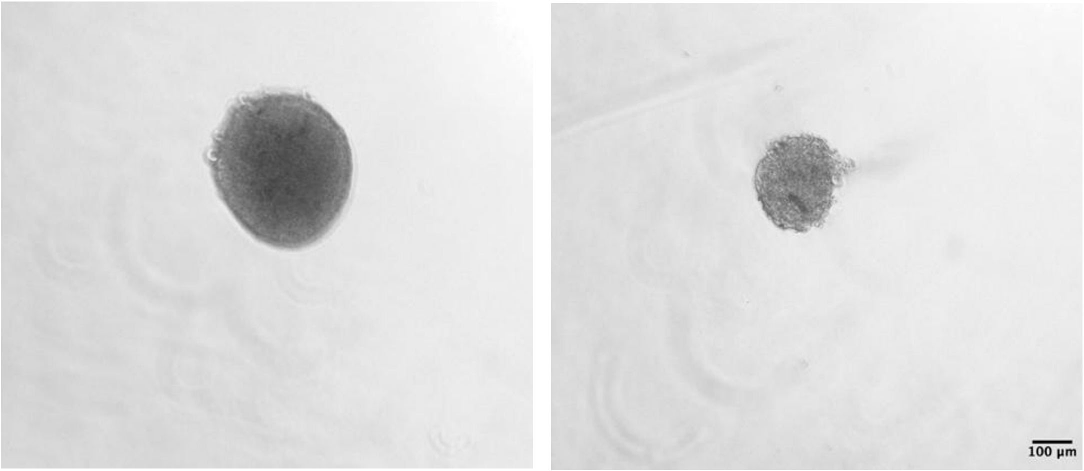

Figure S9. Collagen Supplementation Has Variable Effects. Cell spheroids were supplemented with 1µL of 0.05 mg/mL collagen into the cell suspension before spheroid formation then stained with Von Kossa for calcium. Repeat trials of this experiment revealed variable incorporation of collagen and variable sized aggregates. There was no statistically significant difference in cell calcification after 4 or 7 days when compared to non-supplemented control spheroids.

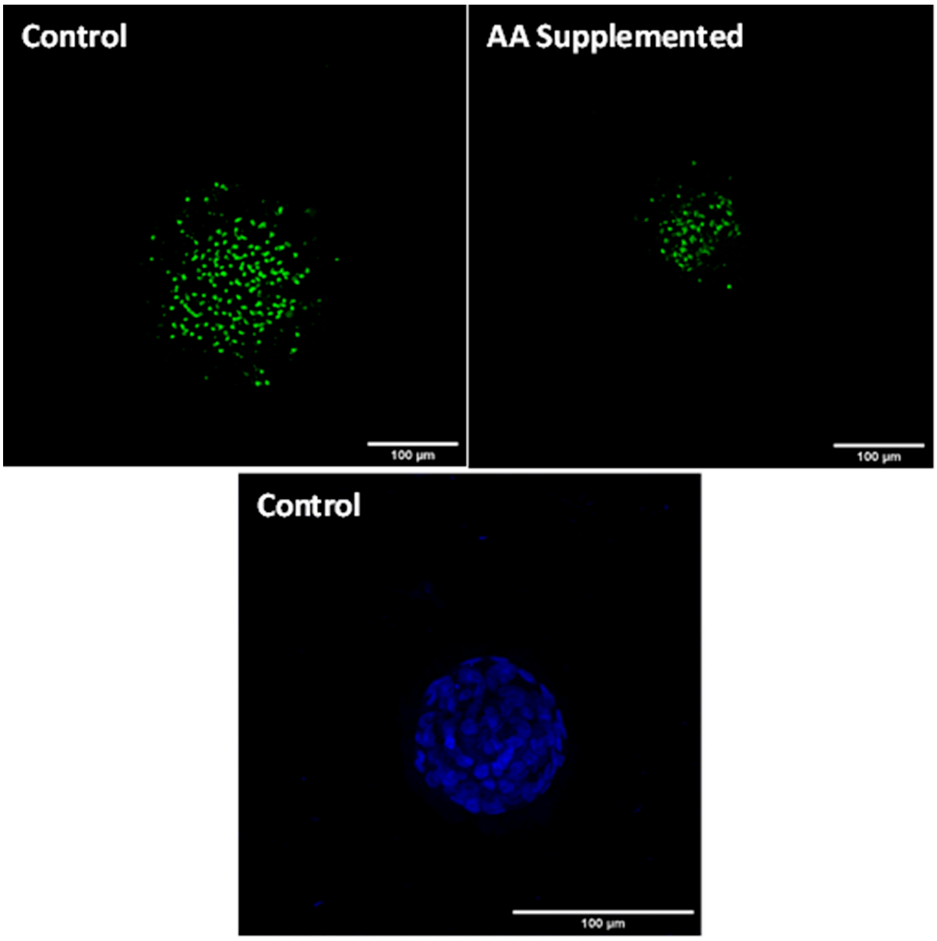

Figure S10. Confocal Images taken of PAVIC 3D spheroid. Spheroids were stained with Propidium Iodide (Top) and Hoechst (Bottom) to better understand the location of the cells as well as where apoptosis is occurring. From the Propidium Iodide staining we were able to confirm that cellular death is occurring in the center of the aggregates with little to no cell death along the outer edges. The Hoechst staining revealed that the cells were rather evenly spread around the spheroid with less live cells in the center of the spheroid.
